# Esophageal submucosal gland duct adenoma: a case report and pooled analysis of demographic differences between eastern and western populations

**DOI:** 10.3389/fonc.2026.1762765

**Published:** 2026-07-02

**Authors:** Yuhua Chen, Yafei Zhang, Liuqing Ge

**Affiliations:** 1 Department of Gastroenterology, Zhongnan Hospital of Wuhan University, Wuhan, China; 2 Hubei Clinical Center & Key Laboratory of Intestinal and Colorectal Diseases, Wuhan, China

**Keywords:** endoscopic submucosal dissection, esophageal submucosal gland duct adenoma, geographic variation, rare diseases, sex distribution

## Abstract

**Background:**

Esophageal submucosal gland duct adenoma (ESGDA) is an exceptionally rare benign tumor. Classic teaching suggests a predilection for the upper and mid esophagus. This study reports a new case and performs a comparative literature analysis to test for potential geographic variations.

**Case introduction:**

A 61-year-old Asian male was found to have a 1.6 cm submucosal tumor in the distal esophagus during a routine check-up. The lesion was successfully resected via endoscopic submucosal dissection (ESD), with pathological confirmation of ESGDA.

**Methods:**

We conducted a pooled analysis of 20 well-documented ESGDA cases from the literature. Cases were stratified into an Eastern cohort (n=10) and a Western cohort (n=10) based on the first author’s affiliation. Demographic and tumor characteristics were systematically compared. Given the small sample size, Fisher’s exact test was applied for categorical variable comparisons.

**Results:**

Striking differences were observed. The Eastern cohort exhibited a profound male predominance (9:1 male-to-female ratio), whereas the Western cohort had a balanced sex distribution (1:1). Fisher’s exact test for this comparison yielded a two-sided P value of 0.141. Tumor location also differed dramatically: 100% of Eastern cases were located in the distal esophagus/gastroesophageal junction, compared to only 40% of Western cases, with 60% of Western cases found in the mid or upper esophagus, a pattern not seen in the Eastern cohort (Fisher’s exact test, P = 0.011). Mean age and tumor size were similar between groups.

**Conclusion:**

The clinical presentation of ESGDA demonstrates notable geographic patterns. In this analysis, Eastern cases presented exclusively in the distal esophagus with a strong male predominance, while Western cases showed a broader anatomical distribution and a balanced sex ratio. These descriptive findings suggest potential population-specific differences. Clinicians should be aware of these patterns, and future studies with larger sample sizes are warranted for confirmation.

## 1 Introduction

Esophageal submucosal gland duct adenoma (ESGDA) is a rare, benign tumor originating from the ducts of the esophageal submucosal glands (1, 2). Since its initial description, understanding of this entity has been based largely on individual case reports, which traditionally emphasize its occurrence in the upper and mid esophagus, reflecting the distribution of submucosal glands. However, a growing number of reports from Eastern Asia, particularly China, suggest that the clinical profile of ESGDA may not be uniform across different populations (3, 4). Aggregating data without geographic stratification may obscure significant epidemiological patterns, leading to potential diagnostic oversight.

We herein report a new case of ESGDA in an 61-year-old male located at the gastroesophageal junction. Furthermore, we perform a pooled analysis of 20 meticulously documented cases from the literature. The primary aim of this study is to systematically compare the clinicopathological features of ESGDA between patients from Eastern and Western populations to identify and characterize any potential differences.

## 2 Case description

### 2.1 Clinical data

**Figure 1:**
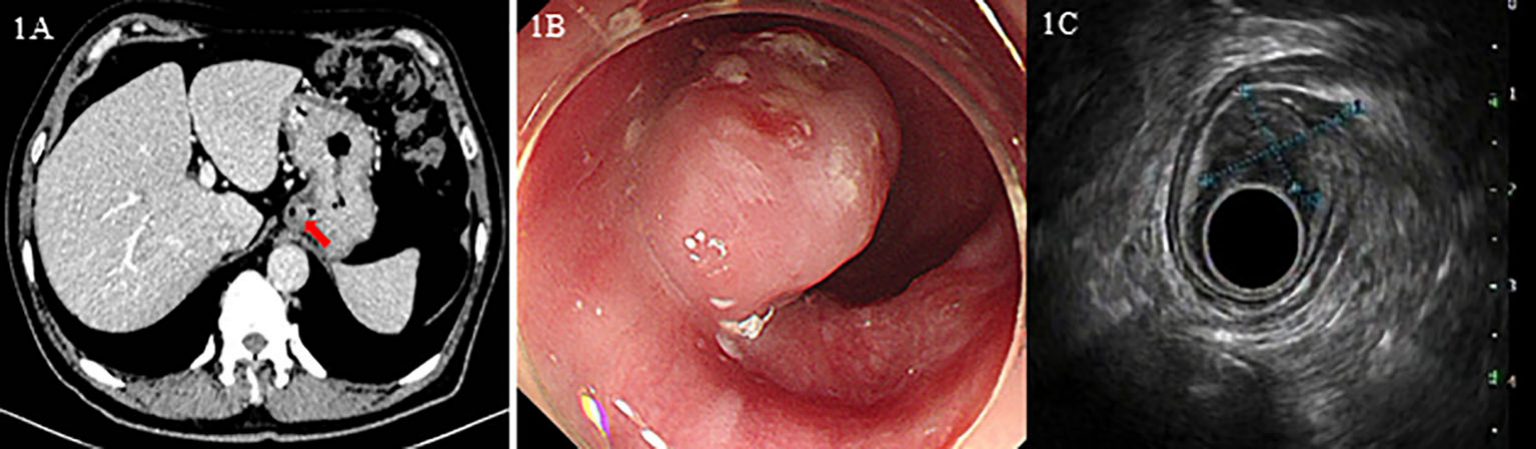
Multimodal imaging with CT, endoscopy, and endoscopic ultrasound(EUS) **(A)** Slight localized wall thickening of the lower esophagusis seen in the cross-sectional view of thoracicand abdominal enhanced CT; **(B)** shows the mucosal bulge near the cardia of the esophagus: **(C)** EUS revealed a well-demarcated, homogeneous hypoechoic mass originating from the submucosa.

A 61-year-old asymptomatic man was referred for assessment of a submucosal lesion in the distal esophagus, detected incidentally during screening endoscopy. His past medical history was significant for hypertension, diabetes mellitus, and sinus tachycardia. Physical examination and laboratory data on admission were unremarkable. Contrast-enhanced CT imaging demonstrated wall thickening in the distal esophagus with heterogeneous enhancement (**Figure 1A**). Endoscopic evaluation showed a 18 × 15 mm protruding lesion with congested and eroded surface mucosa, which was firm to palpation (**Figure 1B**). EUS revealed a well-demarcated, homogeneous hypoechoic mass originating from the submucosa, measuring 16 × 11 mm (**Figure 1C**). Preliminary diagnoses considered were leiomyoma, GIST, and fibrovascular polyp. Endoscopic submucosal dissection was performed for diagnostic and therapeutic purposes.

### 2.2 Pathological examination

**Figure 2:**
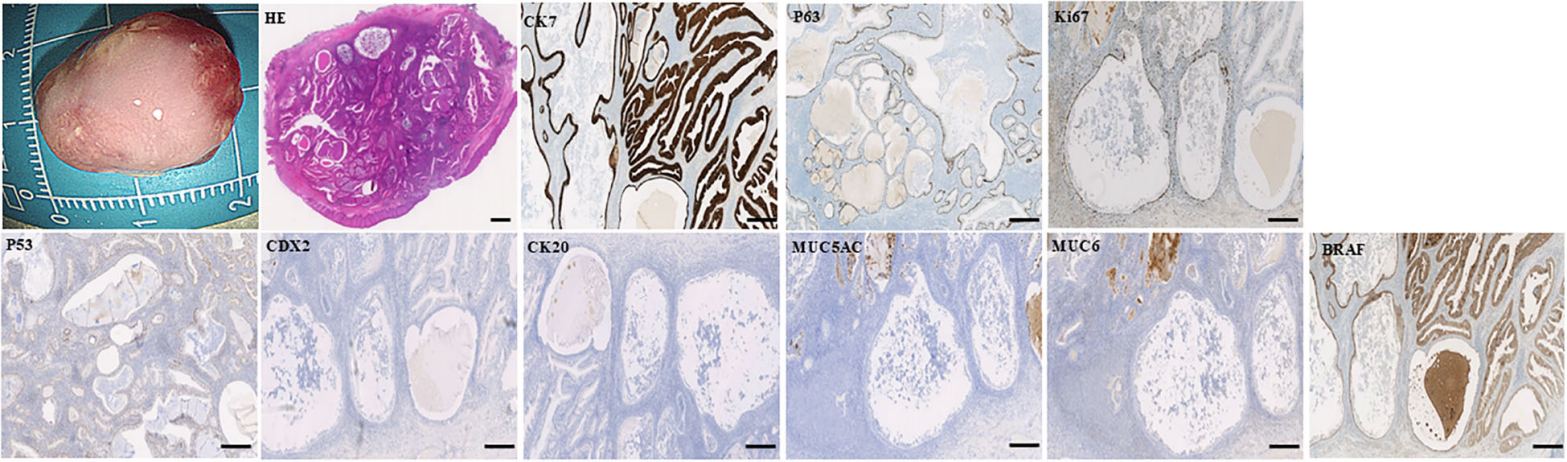
Show the endoscopic resection specimen and histopathology The resected specimen with a diameter of approximately 2cm; show the histology of the case. Low (hematoxylin-eosin,×20)and intermediate (hematoxylin-eosin,×100); The luminal cells expressed CK7, while the myoepithelial layer showed strong nuclear positivity for p63. The benign nature was supported by a low Ki-67 proliferation index (<2%) and a wild-type p53 staining pattern. Notably, the tumor cells were negative for CDX2, MUC5AC, MUC6, and CK20. BRAF V600E immunohistochemistry was performed and yielded a positive result.

Histopathological examination confirmed the diagnosis of a benign submucosal gland duct adenoma. The lesion was characterized by proliferating ductal structures lined by a characteristic bilayered epithelium: an inner layer of luminal columnar cells and an outer layer of myoepithelial cells, recapitulating the morphology of normal ductal anatomy. No significant cytological atypia or increased mitotic activity was observed. Immunohistochemical staining definitively demonstrated this bilinear differentiation. The luminal cells expressed CK7, while the myoepithelial layer showed strong nuclear positivity for p63. The benign nature was further supported by a low Ki-67 proliferation index (<2%) and a wild-type p53 staining pattern. Notably, the tumor cells were negative for CDX2, MUC5AC, MUC6, and CK20, effectively excluding alternate diagnoses such as metastatic adenocarcinoma or mucoepidermoid carcinoma. Given its histomorphological similarity to salivary gland analogues, BRAF V600E immunohistochemistry was performed and yielded a positive result, a finding recently associated with this entity (**Figure 2**).

In conclusion, the combined histologic and immunohistochemical profile—featuring a benign bilayered ductal proliferation, a low proliferation index, and BRAF V600E positivity—is diagnostic of esophageal submucosal gland duct adenoma.

## 3 Literature review and comparative analysis

### 3.1 Methods

A pooled analysis was conducted based on a summary table of 19 histologically confirmed EGDA cases compiled from the literature, to which we added our present case, yielding a total of 20 cases for analysis (5). Cases were categorized as “Eastern” if the first author’s affiliation was in China (references 8, 14, 17, 18, 19, including “our case” from the original table; n=10) and “Western” for all other cases (n=10) (5). Data on patient age, sex, tumor location, and size were extracted. Analysis was performed using descriptive statistics.

### 3.2 Results of pooled analysis

#### 3.2.1 Overall cohort characteristics

The analysis included 20 patients (14 males, 6 females) with a mean age of 67.1 years. The most common location was the distal esophagus (60%), followed by the mid esophagus (15%), gastroesophageal junction (15%), and upper esophagus (10%). The mean tumor diameter was 10.6 mm. Endoscopic resection was the primary treatment.

#### 3.2.2 Comparative analysis: east vs. west

The comparative analysis revealed striking differences between the Eastern and Western cohorts, as summarized in **Table 1**.

**Table 1:** Comparative Analysis of Clinicopathological Features of Esophageal Gland Duct Adenoma between Eastern and Western Cohorts.

Feature	Eastern cohort (n = 10)	Western cohort (n = 10)	P-value / statistical remark
Age, years			
Mean ± SD	66.2 ± 11.5	68.0 ± 7.2	> 0.05
Sex, n (%)			
Male	9 (90.0)	5 (50.0)	0.141
Female	1 (10.0)	5 (50.0)	(Fisher’s exact test)
Tumor Location, n (%)			
Distal esophagus / GEJ	10 (100.0)	4 (40.0)	0.011
Mid /Upper esophagus	0 (0.0)	6 (60.0)	(Fisher’s exact test)
Tumor Size, mm			
Mean ± SD	10.5±9.1	11.1 ± 5.2	> 0.05

P-values for categorical variables (Sex, Tumor Location) were calculated using Fisher's exact test (two-sided) due to the small sample size. The P-value for sex distribution (0.141) should be interpreted with caution given the limited sample size, which reduces statistical power to detect a difference, even when a marked descriptive disparity (90% vs. 50% male) is observed.

- Sex Distribution:​ A profound male predominance was observed in the Eastern cohort (90% male), in stark contrast to the perfectly balanced sex distribution in the Western cohort (50% male). Fisher’s exact test applied to this 2x2 contingency table resulted in a two-sided P value of 0.141.
- Tumor Location:​ All Eastern cases (100%) were located in the distal esophagus or GEJ. Conversely, Western cases showed a markedly different distribution, with a majority (60%) occurring in the mid or upper esophagus. Fisher’s exact test for this distribution yielded a P value of 0.011.
- Age and Tumor Size:​ The mean age (Eastern: 66.2 ± 11.5 years; Western: 68.0 ± 7.2 years) and mean tumor size (Eastern: 10.5 ± 9.1 mm; Western: 11.1 ± 5.2 mm) were comparable between the two groups, with no statistically significant differences observed (both P > 0.05, using appropriate tests for continuous data).

## 4 Discussion

This study presents a new case of ESGDA and provides a novel comparative analysis that uncovers notable geographic patterns in its clinical presentation. Our patient—a 61-year-old male with a distal ESGDA—is a quite essential example of the phenotype characteristic of the Eastern population. The analysis reveals two major, and likely interrelated, geographic disparities.

First, the sex distribution​ is dramatically different, with an overwhelming male predominance (9:1) in the East cohort compared to a balanced distribution in the Western cohort (1:1). It is important to note that in our analysis, Fisher’s exact test for this comparison yielded a P value of 0.141. While the descriptive difference is substantial, this P value, derived from the limited sample of 10 cases per group, indicates that the observed disparity did not reach conventional statistical significance in this dataset. This likely reflects the low statistical power inherent to small sample sizes to detect such differences, and the finding warrants investigation in larger cohorts. Second, the finding that 100% of Eastern cases are located in the distal esophagus, in contrast, Western cases showed a broader distribution, with only 40% in the distal/GEJ region and 60% in the mid or upper esophagus. Fisher’s exact test confirmed this difference was statistically significant (P = 0.011).​ This finding directly challenges the classic teaching that ESGDA predominantly occurs in the upper and mid esophagus and suggests that pathogenic factors in Eastern populations may specifically involve the distal esophageal microenvironment.

The reasons for these disparities are unknown but could involve genetic susceptibility, exposure to specific environmental or dietary factors, or a combination thereof. These findings have direct clinical implications. For endoscopists and pathologists in Eastern countries, ESGDA must be a primary diagnostic consideration for submucosal lesions in the distal esophagus, particularly in male patients. In Western populations, the diagnostic spectrum should remain broad, encompassing the entire esophagus and both sexes equally. The limitations of our study include its retrospective nature and the potential for publication bias. However, the strength and consistency of the observed differences are compelling and highlight the need for geographic stratification in the understanding of rare diseases.

## 5 Conclusion

In conclusion, our pooled analysis of 20 ESGDA cases demonstrates distinct geographic clinical patterns. Eastern cases in this series presented exclusively in the distal esophagus/GEJ, whereas Western cases showed a broader anatomical distribution, a difference that was statistically significant. A strong descriptive male predominance was observed in the Eastern cohort, though this difference did not reach statistical significance in our limited sample. These findings suggest that the clinical presentation of ESGDA may not be uniform across populations. Recognizing these potential differences is important for clinical awareness and diagnostic evaluation. Future collaborative studies with larger sample sizes are essential to confirm these patterns and explore their underlying etiologies.

## Data Availability

The raw data supporting the conclusions of this article will be made available by the authors, without undue reservation.
