# Supplementary material for: Esophageal submucosal gland duct adenoma: a case report and pooled analysis of demographic differences between eastern and western populations
Source: Front Oncol. 2026 Jul 2;16:1762765. doi: 10.3389/fonc.2026.1762765 (PMC13373749; doi:10.3389/fonc.2026.1762765)
Supplement: Supplementary file 1 [file DataSheet1.docx]

### **Table S1: Individual Clinicopathological Data of 20 Reported ESGDA Cases**

| **Case No.** | **Author (Reference)** | **Age** | **Gender** | **Tumor Site** | **Ethnicity**  **(Based on Author Affiliation)** |
| --- | --- | --- | --- | --- | --- |
| 1 | Tsutsumi et al. (1990) | 77 | M | Mid esophagus | Western |
| 2 | Takubo et al. (1993) | 58 | F | Mid esophagus | Western |
| 3 | Rouse et al. (1995) | 81 | M | Distal esophagus | Western |
| 4 | Su et al. (1998) | 70 | M | Distal esophagus | Eastern (China) |
| 5 | Agawa et al. (2003) | 71 | M | Distal esophagus | Western |
| 6 | Hayashi et al. (2004) | 60 | F | Upper esophagus | Western |
| 7 | Chinen et al. (2004) | 60 | M | Distal esophagus | Western |
| 8 | Harada et al. (2007) | 75 | M | Distal esophagus | Western |
| 9 | Shibata et al. (2017) | 66 | F | Distal esophagus | Western |
| 10 | Nie et al. (2016) - Case 1 | 74 | M | Distal esophagus | Eastern (China) |
| 11 | Nie et al. (2016) - Case 2 | 54 | F | Distal esophagus | Eastern (China) |
| 12 | Nie et al. (2016) - Case 3 | 45 | M | Distal esophagus | Eastern (China) |
| 13 | Genere et al. (2019) | 78 | F | Upper esophagus | Western |
| 14 | Yamamoto et al. (2020) | 72 | F | Distal esophagus | Western |
| 15 | Wang et al. (2020) | 70 | M | Gastroesophageal junction | Eastern (China) |
| 16 | Qin et al. (2021) | 53 | M | Distal esophagus | Eastern (China) |
| 17 | Hua et al. (2021) - Case 1 | 63 | M | Distal esophagus | Eastern (China) |
| 18 | Hua et al. (2021) - Case 2 | 65 | M | Distal esophagus | Eastern (China) |
| 19 | Hua et al. (2021) - Case 3 | 75 | M | Gastroesophageal junction | Eastern (China) |
| 20 | New Case (Zhou et al., 2025 / Doc 2)​ | 61 | M | Gastroesophageal junction | Eastern (China) |
